# Deployment and Operation of Outdoor Treatment Tents During the COVID-19 Pandemic

**DOI:** 10.1017/dmp.2020.355

**Published:** 2020-09-11

**Authors:** Keith H.J. Peterson, Erin Joy Cavin Muckey

**Affiliations:** University Hospital, Emergency Department, Newark, New Jersey

**Keywords:** COVID-19, Emergency Preparedness, Pandemic, Triage Tent, Surge Capacity

## Abstract

The coronavirus disease 2019 (COVID-19) pandemic spread throughout the globe, with an alarming amount of new cases daily. To prepare for the inevitable patient surge, 1 hospital set up outdoor triage tents to assist with increased volume. Using the paradigm of space, staff, and stuff, an outdoor treatment area was designed and placed into operation. The patient volume in the treatment tents quickly grew with a 1-d max volume of 88 patients. Through the end of May 2020, a total of 2473 patients were seen and evaluated. As COVID-19 continues to spread and new areas of the United States and the world see spikes, it is imperative for the hospitals that previously dealt with a surge to disseminate the best practices they have learned during the pandemic.

On March 11, 2020, the Director-General of the World Health Organization declared coronavirus disease 2019 (COVID-19) to officially be a pandemic.^1^ Four days later, on March 15, 2020, one tertiary teaching hospital in Northern New Jersey, with the assistance of the New Jersey Emergency Medical Services Task Force (NJEMSTF), erected its first outdoor treatment tent. On that date, there were 1678 cases and 41 deaths in the United States.^2^

The tent was placed under the control of the emergency department (ED) as an arrival and treatment area for stable patients with respiratory symptoms, in preparation for an expected surge in patient volume. The staff and leadership of the ED were tasked with designing and implementing the triage tents to maximize the benefit for departmental operations and the provision of safe and efficient patient care. It was determined that the best use of space would be as a screening area for patients presenting with possible COVID-19 symptoms. Knowing a patient surge was about to occur, the ED prepared by using the paradigm of space, staff, and stuff.^3^

## REQUIRED RESOURCES

### Space

**FIGURE 1:**
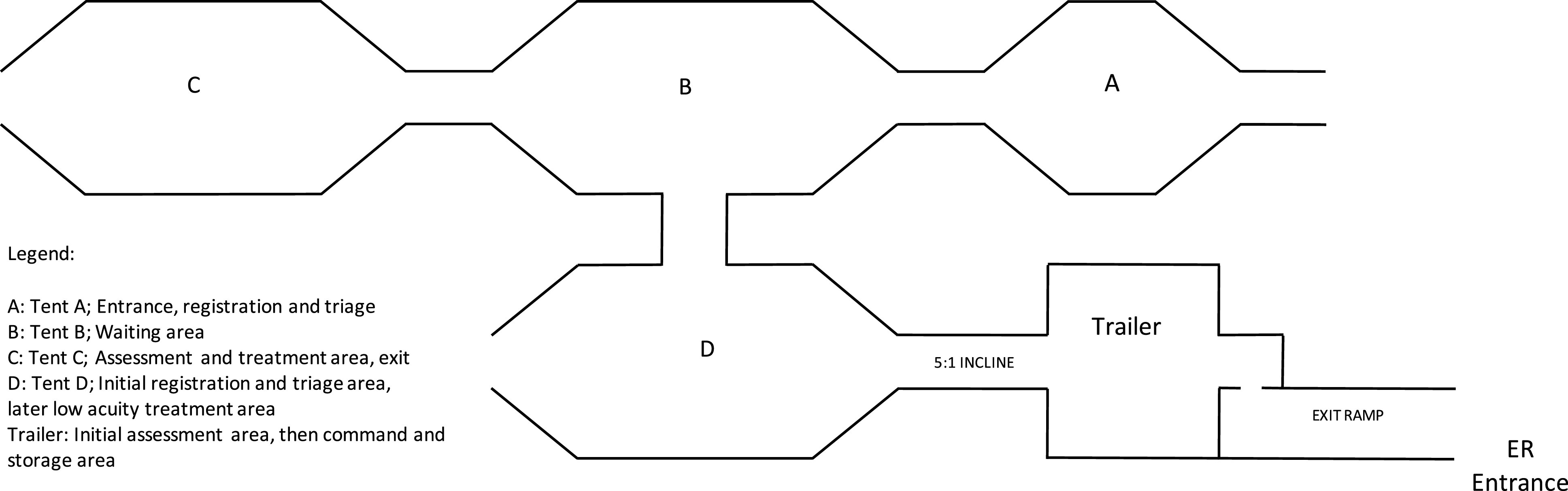
Tent layout

The first tent to be set up for use was a Gate Keeper 1935 (Western Shelter, Eugene, OR), known as tent D, see Figure 1. This tent model provided 53 m^2^ of treatment space. An M-Rets 24 (Western Shelter, Eugene, OR) trailer was attached to the tent and was connected by means of a 4.5-m ramp with 1:5 slope, providing an additional 31.2 m^2^ of space. This area served as the jump-off point for the COVID-19 triage tents. During the preparation phase of operations, plans were created around this space. Once in the operational phase, it was quickly determined that additional space would be required.

With the assistance of the NJEMSTF, 3 additional tents were set up, 2 additional Gate Keeper 1935s, tents B and C, and 1 Gate Keeper 20 (Western Shelter, Eugene, OR) with 26.6 m^2^ of treatment space, tent A. All 4 tents and the trailer were connected by vestibules, each measuring 5.2 m^2^, creating 1 enclosed treatment area. A decision was made to have the trailer function as a command and storage area and would not be a patient care area. The new complex provided 232.4 m^2^ of treatment and command space.

### Staff

Three groups of hospital staff were required to operate the tent: admitting, nursing, and providers. It was determined that 2 staff members from admitting would ensure patients could be promptly entered into the system and fully registered. ED nurses would staff the tent to triage patients safely. Advanced practice nurses (APN) from the ED observation unit would serve as providers. These APNs were available as the observation unit had been converted to an inpatient unit to meet growing hospital demands. As the pandemic increased and outpatient clinics were closed, the APNs and physician assistants (PAs) that staffed those clinics were used to supplement the observation staff.

Following the hospital emergency operations plan, a labor pool was formed. Staff members assigned to the labor pool served in various roles, such as runners who would help gather supplies and send samples to the lab. Additional help arrived on April 14th when the Army Urban Augmentation Medical Task Force 332-1 arrived with 85 soldiers.^4^ The task force provided the aid of military nurses, registration personnel, and APNs and PAs to the tent. Once the soldiers were oriented to tent operations, they provided a large amount of the staff that was working in the tents. This allowed hospital employees to return inside to assist with the acutely ill. The army supplemented hospital staff until May 20th, fortunately by that time the surge had abated, and hospital personnel was able to staff the tents.

### Stuff

The gathering of supplies and equipment provided the most extensive logistical need for the setup of the tents. Building the tents from scratch required gathering all perceivable items, including the smallest things such as pens, paper, and garbage cans.

Medical supplies were the most straightforward items to gather. Required supplies included portable vital sign machines, blood glucose monitors, disposable stethoscopes, and nasopharyngeal swabs for COVID-19 testing. Adult and pediatric code carts were obtained in case of any emergencies. For a brief period during the height of the pandemic, tent D was used as a low acuity treatment area, and phlebotomy and IV supplies were added to the tent, and it was hooked up to an oxygen trailer where mildly hypoxic patients received oxygen.

Personal protective equipment was required in the tent due to the highly contagious nature of COVID-19. N95 respirators would not be kept in the tent and would be distributed from within the hospital. The decision was made to ensure that staff received the proper N95 that they were fit tested to wear. Isolation gowns and eye protection were kept within the tent to ensure easy access. A large number of gloves, cleaning products, and hand sanitizer were obtained.

Tables and chairs were obtained and used to set up the assessment, waiting, and treatment areas. Patient privacy screens were erected and critical to ensuring the privacy of the patients. Information technology (IT) staff set up 8 desktop computers, 4 mobile workstations, scanners for patient registration, and printers that could also print prescriptions. IT wired the tent that allowed for both ethernet and Wi-Fi availability. A large generator, provided by the NJEMSTF, provided electricity to the tents. Eventually, large sections of the tent were hardwired to hospital power by the hospital electricians. Multiple heating/ventilation/air conditioning (HVAC) units accomplished temperature control. A list of required material is available as an online supplement.

## PATIENT FLOW

An organization can have an infinite amount of space, staff, and stuff, but without well-established patient flow, proper care cannot be accomplished. A flow was created, and minor changes were made as the tents became operational. Ambulatory patients that arrived at the ED were first screened at the ED entrance. If the patient arrived without a mask, they were issued a mask at this time as masks were required both within the tent and inside the main hospital. Patients with respiratory complaints and stable oxygen saturation were redirected to the tent and entered through tent A, which was adjacent to the ED entrance. At the entrance there was a hand washing station; staff would direct all patients to wash their hands with soap and water. Once inside, patients were entered into the electronic health record by registration staff, triaged by an ED RN, and then queued in the waiting area located in tent B, which was designed to space patients by at least 6 ft. An APN or PA would then perform a medical screening exam (MSE), in tent C. During this exam, the provider would decide if the patient required COVID-19 testing based on Centers for Disease Control criteria. To maintain 1-directional flow, the APN or PA would provide discharge teaching and any needed prescriptions. Before exiting, the patients would be directed to an additional handwashing station located at the exit. If, at any point it was decided the patient was acutely ill and not appropriate to be evaluated in the tent, the triage nurse would call the charge nurse in the ED and escort the patient to the ED. These patients were transported by means of wheelchair into the ED, a stretcher was located within the tent and fortunately was never required.

## INFECTION CONTROL

While the Gate Keepers are capable of becoming negative pressure, it was decided not to make the tents negative pressure. The first reason is that the command trailer system would need to be disconnected. Second, and perhaps more important, is that with the large number of expected patients, multiple doors would be continually opening and closing, defeating the purpose.

All staff in the tent were told that preventing cross-contamination and cleaning was everyone’s job. Registration would wipe down chairs in their area between every patient, as well as any items the patient touched. The nurse was responsible for cleaning their triage are and disposable equipment was used. The runners would clean the chairs in the waiting area between each patient, and the APNs and PAs would clean their area between patients.

## PATIENT VOLUME

On March 24, 2020, at 12:15 pm, tent D and the trailer opened for patients. It was opened until 4:00 pm, and 12 patients were evaluated, a soft opening of sorts. The next day, the tent was opened from 8:30 am to 4:00 pm, and 41 patients were seen, and 42 were seen the following day. This quick rise in patient volume is what necessitated the need for the 3 additional tents. The hours established for tent use were set to be 7 d a wk from 08:00 am to 6:00 pm.

The volume of the tents quickly spiked in the second full week of operation. The week of April 5th saw 528 patients evaluated in tents and contained the busiest single day on April 9th, with 88 patients.

From that week on, the tents would have a steady decline in volume, with a sharp decline beginning to present halfway through May. Through the end of May, the total patient volume was 2,473. Of those patients 79.7% were tested for COVID-19, with a positivity rate of 28.1%, see Table 1. As the volume continued to downtrend in the area, the decision was made to leave the triage tents in place. They remained a triage area for those presenting with COVID-19 symptoms and were also being used to perform preadmission testing as elective surgeries restarted. The tents ceased operations all operations on July 1, 2020, as of the writing of this article the structures remain in place.

**TABLE 1:** Patient Volume and Testing

Week of	Patient Volume	Tested for COVID-19	Positive for COVID-19	Percent Positive
3/24/20*	172	27	15	8.72%
3/29/20	306	170	102	33.33%
4/5/20	528	423	211	39.96%
4/12/20	351	317	135	38.46%
4/19/20	303	286	95	31.35%
4/26/20	267	241	64	23.97%
5/3/20	223	205	33	14.8%
5/10/20	142	136	20	14.08%
5/17/20	125	114	17	13.6%
5/24/20^^+^^	56	52	3	5.36%
5/31/20^*^	0	N/A	N/A	N/A

* Not a full week.

+ Closed Memorial Day.

## IDENTIFIED ISSUES

It is often said that no plan survives first contact with the enemy, and COVID-19 truly is an enemy. By and large, there were no significant issues during the operation of the triage tents. The most substantial issues occurred when operations moved from 1 tent to the multi-tent complex. The planned flow was not ideal and had to be reversed in the middle of operations. When the operations consisted of the single tent and the trailer, patients entered Tent D and were examined and exited on the trailer side. When the additional tents were put into operations, the entrance was at tent C and the exit at tent A. It was quickly seen this was not ideal based on the new set-up and the equipment and flow needed to be reversed.

The tents were always well-staffed; however, due to hospital demands, there was not enough staff to allow for 24/7 coverage. One issue that arose was that different staff members were staffing the tents daily, which necessitated repeated just-in-time training by the RN coordinator.

Another repetitive issue was the generator and HVAC operations. The units were dated and had been in storage for some time. In total, 3 different generators were used, and the HVACs required constant maintenance during operations. The HVAC units had difficulty in maintaining temperature control when the outside temperature was 4.5°C or lower. A diesel-powered external heater was used to supplement the HVACs. As the days grew warmer, the opposite issue occurred. It was found that the HVAC units would internally freeze if left in air conditioning mode overnight and shut down. It was quickly determined that by raising the temperature of the system overnight, that issue could be bypassed.

## CONCLUSIONS

The COVID-19 pandemic appeared with a sudden force that took the world of health care and the entire planet off guard. Many institutions had to quickly think out of the box to meet the sudden surge of extremely critically ill patients. The current situation of COVID-19 is changing every day, and there are now spikes in cases both nationally and internationally. With that, best practices learned during the first months of the outbreak can lead to improved patient outcomes in the future.

## Supplementary material

For supplementary material accompanying this paper visit http://dx.doi.org/10.1017/dmp.2020.355. click here to view supplementary material
