# Supplementary material for: Deployment and Operation of Outdoor Treatment Tents During the COVID-19 Pandemic
Source: Disaster Med Public Health Prep. 2020 Sep 11:1–4. doi: 10.1017/dmp.2020.355 (PMC7711347; doi:10.1017/dmp.2020.355)
Supplement: Supplementary file 1 [file S1935789320003559sup001.docx]

Online Supplement 1. Supply List

The following list of items were required for the operation of the tents. Certain items do not have a quantity listed as they required constant resupply.

| Item | Quantity |
| --- | --- |
| Folding Tables | 7 |
| Folding Chairs | 30 |
| Desktop Computers | 8 |
| Mobile Computers | 4 |
| Patient Privacy Screens | 8 |
| Garbage Cans | 10 |
| Garbage Bags |  |
| Adult Code Cart | 1 |
| Pediatric Code Cart | 1 |
| Defibrillator | 1 |
| Intubation Box | 1 |
| Printers | 3 |
| Printer Paper |  |
| Prescription Blanks |  |
| Armband Printers | 2 |
| Patient Label Printer | 1 |
| Scanner | 1 |
| Emergency Backup Lights | 8 |
| Specimen Refrigerator | 1 |
| HVAC Units | 3 |
| Diesel Heater | 2 |
| Handwashing Station | 2 |
| Direction Signs |  |
| EMTALA Signs | 3 |
| Patient Bill of Rights Signs | 3 |
| Phones | 4 |
| Video Translator | 2 |
| Secure two-way radios | 3 |
| Field cots | 10 |
| Wheelchair | 1 |
| Hospital Stretcher | 1 |
| Paper Sheets |  |
| Vital Sign Machine | 2 |
| Mounted Hand Sanitizer | 4 |
| Sanitizer Refills |  |
| Gloves (All Sizes) |  |
| Disinfectant Wipes (Various) |  |
| Isolation Gowns |  |
| Eye Protection |  |
| Disposable Blood Pressure Cuffs |  |
| Disposable Pulse Oximetry |  |
| Oral Thermometer | 4 |
| Thermometer Prob Cover |  |
| Stop the Bleed Kit | 1 |
| Ice Packs | 5 |
| 4x4 Gauze Pads | 5 boxes |
| COVID-19 Specimen Swabs |  |
| Blood Glucose Monitor and Supplies | 1 |
| IV Start Kit | 20 |
| 20ga IV | 1 Box |
| Saline Flushes | 1 Box |
| IV Tubing | 20 |
| 1000ml 0.9% Normal Saline | 15 |
| Nasal Cannulas | 20 |
| Nonrebreather Mask | 20 |
| Oxygen Tubing | 30 |
| Vacutainer | 2 Boxes |
| Vacutainer Push Button Collection Set 23ga | 1 Box |
| Vacutainer Push Button Collection Set 21ga | 1 Box |
| Alcohol Prep Pads | 2 Boxes |
| Bandages | 4 Boxes |
| Blood Tubes |  |
| K2 EDTA Additive | 2 Packs |
| SST Additive | 2 Packs |
| PST Gel and Lithium Heparin Additive | 2 Packs |
| Buff Na Citrate Additive | 2 Packs |
| Sodium Fluoride Potassium Oxalate Additive | 2 Packs |
